# A Flavor of the Future of GI Endoscopy—New Solutions Shape the Field of Modern Gastrointestinal Care

**DOI:** 10.3390/cancers13123007

**Published:** 2021-06-16

**Authors:** Anastasios Koulaouzidis, Wojciech Marlicz, George Koulaouzidis

**Affiliations:** 1 Department of Public Health, Pomeranian Medical University, 71-252 Szczecin, Poland; 2 Department of Gastroenterology, Pomeranian Medical University, 71-252 Szczecin, Poland; marlicz@hotmail.com; 3 Department of Biochemical Sciences, Pomeranian Medical University, 71-252 Szczecin, Poland; koulaou@yahoo.co.uk

Gastrointestinal (GI) cancers remain high on the list of the leading causes of death worldwide; however, the recent emergence of new and ongoing global healthcare threats, such as the COVID-19 pandemic, has led to a temporary reduction in elective endoscopic procedures [1] and, consequently, to a temporary deferral in GI cancers diagnosis [2]. Innovative ideas in prioritizing referrals and/or utilizing new or existing non-contact (or limited), aerosol-free diagnostic techniques and modalities, together with the use of appropriate personal protective equipment, attempt to provide some resolution to this new reality and they are here to stay [1,3,4]. Furthermore, the associated impact in treatment and patients’ outcomes call for the renewal of protocols, artificial intelligence (AI) and telemedicine [5,6], repurposing, or even the emergence of new drugs and drug delivery platforms [7] for more precise and flexible management. In this Special Issue (SI) of *Cancers*, experts in this field will review the current approaches for diagnosing and managing patients with the spectrum of GI cancers, and suggest innovative solutions fit for the diagnosis and (eventually) the management of these cancers in light of the new challenges and abundance of innovations [1].

The SI is a showcase of a fine collection of articles that underpin the continuing research and innovation approaches in GI endoscopy. In the Global Burden, Risk Factors, and Trends of Esophageal Cancer [8]: An Analysis of Cancer Registries from 48 countries, Huang et al. examine the global burden, risk factors, and trends of esophageal cancer based on age, gender, and histological cancer subtype. Esophageal cancer is the seventh most common cancer globally. Using data retrieved from cancer registries databases from 48 countries in the period 1980–2017, the authors evaluate temporal patterns of incidence and mortality by average annual per cent change (AAPC) using joinpoint regression. Furthermore, they examine associations with risk factors using linear regression. It comes as no surprise that the highest incidence esophageal cancer was observed in Eastern Asia. However, the highest incidence of adenocarcinoma (AC) was found in the Netherlands, the United Kingdom, and Ireland. A higher AC/squamous cell carcinoma (SCC) incidence ratio was associated with a higher prevalence of obesity and elevated cholesterol. Huang et al. note an incidence increase (including AC and SCC) in some countries, with the Czech Republic (female: AAPC 4.66), Spain (female: 3.41), Norway (male: 3.10), Japan (female: 2.18), Thailand (male: 2.17), the Netherlands (male: 2.11; female: 1.88), and Canada (male: 1.51) showing the most significant increase. The countries with increasing mortality included Thailand (male: 5.24), Austria (female: 3.67), Latvia (male: 2.33), and Portugal (male: 1.12). The authors conclude that although the incidence of esophageal cancer shows an overall decreasing trend, an increasing trend was observed in some countries with high AC/SCC incidence ratios. It is essential to closely monitor and slow down the growing obesity and metabolic syndrome rates, which are the critical risk factors for adenocarcinoma. Furthermore, the authors advise that with more advanced, flexible and less invasive technology, population-based targeted screening endoscopy would be recommended for high-risk individuals. The growing reflex response to the COVID-19 pandemic has seen several adaptations in the delivery of care from organized healthcare systems, including the use of capsule endoscopy [1,3], Cytosponge [9] and EsoGuard [10], and there is a sense that these changes are here to change and involve ever further.

In another paper of this SI, Verra et al. present their work on a robotic-assisted colonoscopy platform with a magnetically actuated soft-tethered capsule [11]. Almost two million people are newly diagnosed with colorectal cancer (CRC) every year, with a devastating impact on them and their families. Despite offering a prime paradigm for screening, wider adoption of CRC screening is hampered by reluctance due to widespread perceptions of invasiveness and—often unjustified—fears for discomfort and pain with the conventional colonoscopy platforms [12]. Verra et al., supported by the European Commission within the framework of the H2020 European Endoo Project (G.A.: 688592) and by Ministero dell’Istruzione, dell’Università e della Ricerca (MIUR) under the programme “Dipartimenti di Eccellenza ex. L.232/2016” to the Department of Surgical Sciences, University of Torino, Italy, aimed to develop a novel colonoscopy platform. The platform consists of an active locomotion soft-tethered capsule, which, unlike its wireless counterpart, offers both diagnostic and therapeutic capabilities. Capsule navigation is achieved via a closed-loop interaction between two permanent magnets, enhanced by accurate localization [13]. Verra et al. detailed ex-vivo tests that show a 100% success rate in operating channel and target approach tests. Progression of the endoscopic capsule was feasible and repeatable, and interaction forces were lower if compared to conventional colonoscopy (e.g., 1.17N vs. 4.12N). The polyp detection rates were comparable between groups (91% vs. 87%, colonoscopy and Endoo, respectively). The authors conclude that the Endoo colonoscopy platform allows smoother navigation than conventional colonoscopy, providing comparable operational features. If confirmed in clinical trials, it may represent a valuable and novel screening tool for CRC, an area that has been affected by the unavoidable delays caused by the pandemic [2,3,14].

The SI is not short of high-quality reviews in the field of GI endoscopy. Finocchiaro et al. provide their elaborate and highly pictorial revision on the current and future perspectives in training simulators for GI Endoscopy [15]. Conventional GI endoscopic techniques are technically demanding and require visual–spatial skills and significant hands-on experience to master the skill. Furthermore, there is lately a renewed interest in endoscopy-related injuries (ERI) as a possible cause of occupational disease, which can jeopardise precarious balances in the international delivery of GI endoscopy [16,17]. Endoscopic simulators represent a good solution to allow clinicians to practice in pre-clinical scenarios. From the first endoscopy mannequin, developed in 1969, several simulation platforms have been developed, ranging from purely mechanical systems to more complex mechatronic devices and animal-based models [18]. Considering the recent advancement of technologies (e.g., AI, augmented reality, robotics), simulation platforms can now reach high levels of realism, representing a valid and smart alternative to standard trainee/mentor learning programs. Finocchiaro et al. offer a broad view of the technology available for GI endoscopy training, including platforms currently on the market, and the relevant advancements in this research and application field. Additionally, new training needs and new emerging technologies are discussed to understand where medical education is heading.

A high-quality negative screening colonoscopy was related to diminished CRC incidence and mortality for up to 17.4 years [19]. However, in real life, poor visualization frequently alters colonoscopy quality, followed by referrals to perform either CT colonography (CTC) or colon capsule endoscopy (CCE) as an alternative examination. Uncertainty frequently shadows medical practitioners’ decision making regarding future diagnostics following incomplete colonoscopies. In this SI, Deding et al. presented the results of their systematic review with a meta-analysis aiming to compare CTC and CCE, performed after incomplete colonoscopy, regarding completion rate, sensitivity and diagnostic yield (DY) for polyps [20] The CCE completion rate ranged between 65 and 93% (a pooled estimate of 0.76); when defined as a complete colonic view, between 75 and 98% (a pooled estimate of 0.90). In contrast, the completion rate of CTC varied from 86 to 100% (a pooled estimate of 0.90). The DY of CCE varied between 28 and 44% (a pooled estimate of 0.37), while that of CTC ranged from 4% to 33% (a pooled estimate of 0.10). Further, DYs were reported according to polyp sizes, as follows: any size, >5 mm and >9 mm. Deding et al. found that the DY of CCE for polyps of any size was four times higher than CTC and concluded that CCE is superior to CTC, particularly when diagnosing small polypoid colonic lesions. CTC may be more suitable for larger polyps. Although the completion rate of CTC was superior to that of CCE, the complete colonic view rate of CCE was 90%. These findings are important as CCE might be the preferable non-invasive procedure, without the risk of radiation, with a potential to perform it in the privacy of patients’ remote locations [1,20]. Finally, as reported by authors, CCE vs. CTC following incomplete colonoscopy was not inferior to CTC in polyp detection. As the evidence for CCE is still scarce, the authors call for randomized studies exploring the role of CCE with or without CTC following incomplete colonoscopy.

Also, in this SI, Marlicz et al. aimed at providing a timely review of robotic gastroscopes and related technologies supporting professional skills in clinical practice [21]. Authors aimed to inspire new technological developments. Their work is up to date, especially in the era of the COVID-19 pandemic and post-pandemic. As the incidence and mortality of upper gastrointestinal cancers in some regions remain high, the need for continuous screening and early cancer detection is essential. However, the high-quality of upper GI endoscopy is strongly dependent on the professional’s skills and other variables, such as the risk of disease transmission (e.g., SARS-CoV-2), procedure-related muscular injuries, risk of depression and work-related burnout, relocation and unavailability of human or material resources, as well as the patient’s willingness to undergo non-invasive diagnostic tests Therefore upper GI robotic endoscopes, more and more frequently equipped with artificial intelligence (AI), can facilitate the process of medical management and validation of the set of so-called auditable key performance indicators. Robotic gastroscopes can promote the practice by reducing the variation and improving the reproducibility of high-quality examination between practicing endoscopists. Examples of such new devices include gastric capsule endoscopes (e.g., UGI motility-actuated capsule gastroscopes, externally actuated capsule gastroscopes), innovative smart devices for upper-GI tract (e.g., tethered capsule-like gastroscopes, wireless capsule gastroscopes) and novel flexible robots for UGI surgery. As stated by the authors, ‘The promotion of AI-assisted and teleoperated robotic gastroscopes will allow more people to have access to an efficient, standardized, and reliable diagnosis and surgery’ [21]. We live in an era of rapid changes and have to take brave advantage of it. With the aid of new technologies, we need to reshape the field of GI endoscopy and diagnosis and make our future brighter.
